# Retraction Note: Conceptualising a channel-based overlapping CNN tower architecture for COVID-19 identification from CT-scan images

**DOI:** 10.1038/s41598-025-85425-0

**Published:** 2025-01-14

**Authors:** Ravi Shekhar Tiwari, Lakshmi D, Tapan Kumar Das, Kathiravan Srinivasan, Chuan‑Yu Chang

**Affiliations:** 1 Artificial Intelligence Engineering, Chadura Tech, Bangalore, India; 2 School of Computing Science and Engineering, VIT Bhopal University, Bhopal, India; 3 School of Information Technology and Engineering, Vellore Institute of Technology, Vellore, India; 4 School of Computer Science and Engineering, Vellore Institute of Technology, Vellore, India; 5 Department of Computer Science and Information Engineering, National Yunlin University of Science and Technology, Yunlin, 64002 Douliu City Taiwan; 6 Service Systems Technology Center, Industrial Technology Research Institute, Hsinchu, Taiwan

Retraction of: *Scientific Reports* 10.1038/s41598-022-21700-8, published online 28 October 2022

The Editors have retracted this Article because it contains a significant number of nonstandard phrases. The Authors have been unable to sufficiently explain their use of these terms. In addition the Authors have been unable to provide the full augmented training dataset upon request. The Editors therefore no longer have confidence in the reliability of this Article.

Authors Ravi Shekhar Tiwari, Lakshmi D, Tapan Kumar Das and Kathiravan Srinivasan disagree with this retraction. Author Chuan-Yu Chang has not responded to correspondence regarding this retraction.

